# Regulator combinations identify systemic sclerosis patients with more severe disease

**DOI:** 10.1172/jci.insight.137567

**Published:** 2020-09-03

**Authors:** Yue Wang, Jennifer M. Franks, Monica Yang, Diana M. Toledo, Tammara A. Wood, Monique Hinchcliff, Michael L. Whitfield

**Affiliations:** 1 Department of Biomedical Data Science, Geisel School of Medicine at Dartmouth, Lebanon, New Hampshire, USA.; 2 Department of Internal Medicine, Northwestern University Feinberg School of Medicine, Chicago, Illinois, USA.; 3 Yale School of Medicine, Section of Allergy, Rheumatology and Immunology, New Haven, Connecticut, USA.

**Keywords:** Autoimmunity, Dermatology, Autoimmune diseases, Bioinformatics, Skin

## Abstract

Systemic sclerosis (SSc) is a heterogeneous autoimmune disorder that results in skin fibrosis, autoantibody production, and internal organ dysfunction. We previously identified 4 “intrinsic” subsets of SSc based upon skin gene expression that are found across organ systems. Gene expression regulators that underlie the SSc-intrinsic subsets, or are associated with clinical covariates, have not been systematically characterized. Here, we present a computational framework to calculate the activity scores of gene expression regulators and identify their associations with SSc clinical outcomes. We found that regulator activity scores can reproduce the intrinsic molecular subsets, with distinct sets of regulators identified for inflammatory, fibroproliferative, limited, and normal-like samples. Regulators most highly correlated with modified Rodnan skin score (MRSS) also varied by intrinsic subset. We identified subgroups of patients with fibroproliferative and inflammatory SSc with more severe pathophenotypes, such as higher MRSS and increased likelihood of interstitial lung disease (ILD). Using an independent cohort, we show that the group with more severe ILD was more likely to show forced vital capacity decline over a period of 36–54 months. Our results demonstrate an association among the activation of regulators, gene expression subsets, and clinical variables that can identify patients with SSc with more severe disease.

## Abstract 2

An association between the activation of regulators, gene expression subsets, and clinical variables identifies systemic sclerosis patients with more severe disease.

## Introduction

Systemic sclerosis (SSc) is a heterogeneous autoimmune disease that results in the production of autoantibodies, skin fibrosis, and internal organ involvement (1); the pattern and severity of skin and organ involvement varies across patients. Clinically, SSc is divided into 2 subtypes based on the extent of skin involvement, including limited cutaneous SSc (lcSSc) and diffuse cutaneous SSc (dcSSc) (2). The lungs, heart, kidney, and other organs may also be involved (1, 3, 4). We have previously identified “intrinsic” subsets of SSc (fibroproliferative, inflammatory, limited, and normal-like) based upon skin gene expression (5–7) that may predict response to therapy (8, 9). Analysis of skin gene expression across cohorts identified interactions between immune and stromal cells that may act as key drivers of SSc pathogenesis in patients with a permissive genetic background (10, 11).

Regulators, either acting at the transcriptional or posttranscriptional level, control the expression of their target gene networks; thus, they contribute to different biological phenotypes and can be proxies for tightly coordinated and regulated pathways (12). However, the regulators that underlie SSc and the association with clinical phenotypes have not been systematically investigated. The goals of these analyses were 2-fold: first, to identify regulators of gene expression, such as transcription factors (TFs) and miRNAs that are enriched in the SSc-intrinsic subsets and, second, to identify regulators that could identify patients with SSc with more severe skin and lung disease. Our reasoning is that the regulators, and the network of genes that they control, may be informative of pathological processes acting in SSc.

**Figure 1:**
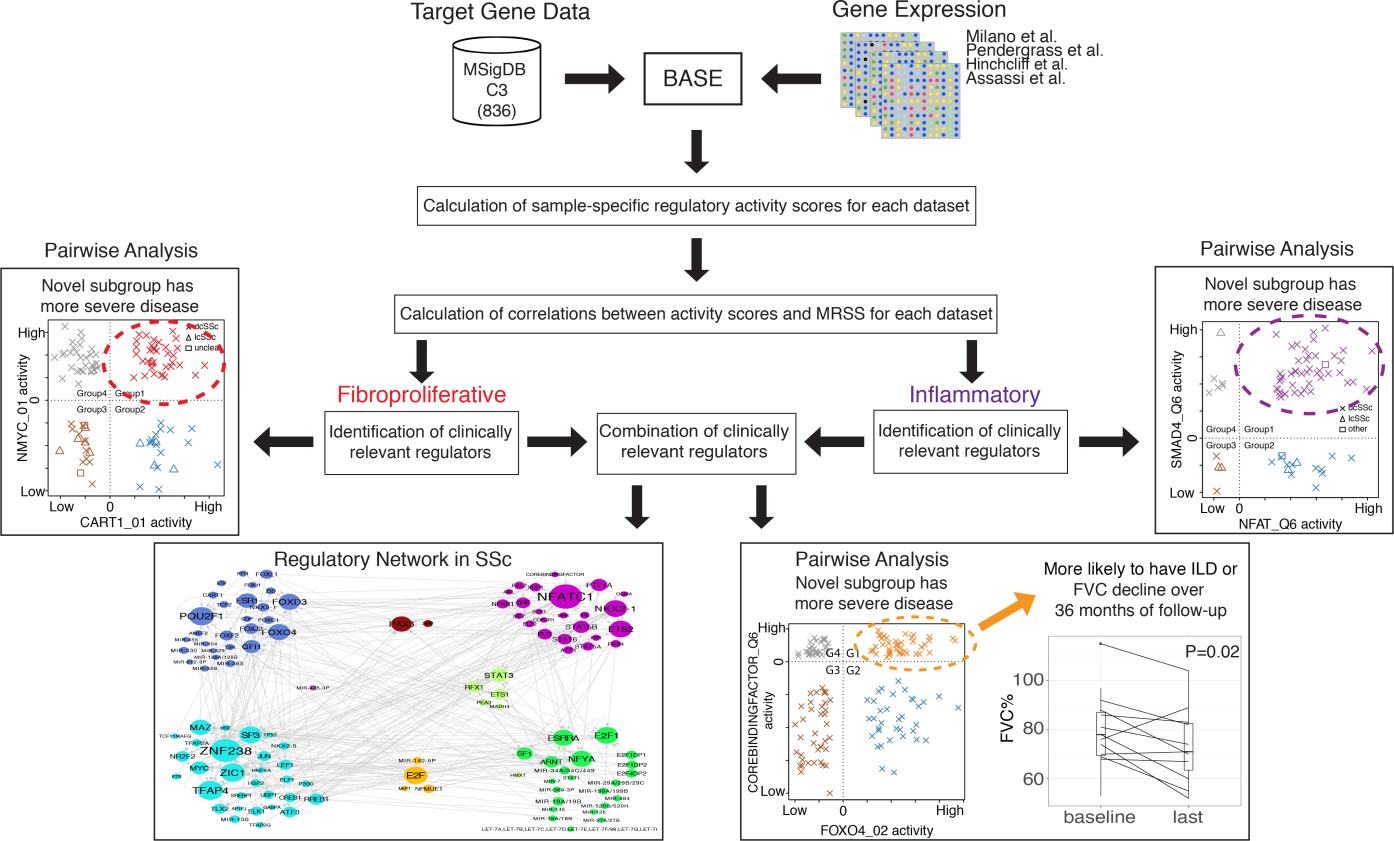
Overview of computational workflow in this study. Briefly, by integrating target gene lists and gene expression of patients with SSc, we calculated sample-specific regulatory activity scores for each data set. Then, by calculating the correlations between activity scores and MRSS, fibroproliferative and inflammatory associated regulators were identified. Using those regulators, we further identified subgroups of patients in a given intrinsic subset, built a regulatory network in the context of SSc, and identified possibly novel subgroups of patients with SSc who are more like to have ILD or FVC decline over 36 months of follow-up.

Herein, we constructed a computational framework to systematically examine the activity of 836 regulators across 431 SSc skin and 35 SSc blood samples using publicly available gene expression data (see Figure 1 for an overview). We characterized each regulator’s target genes and their expression profile to infer the regulator activity scores for each SSc sample using the BASE algorithm (13). Regulator activity scores were correlated with the modified Rodnan Skin Score (MRSS), a common measure of SSc severity, to identify those with activity scores highly associated with severity of skin disease, particularly in fibroproliferative and inflammatory patients. We then built an interaction network using regulators that were substantially associated with MRSS to provide a comprehensive picture of the regulatory interactions within the SSc-intrinsic subsets. Then, subgroups within the fibroproliferative or inflammatory intrinsic subset were identified by using a combination of the activity scores of 2 MRSS-correlated regulators. Further, we found a group of SSc samples from patients with a higher MRSS and significant decline in forced vital capacity (FVC) over 36 months of follow-up.

## Results

### Regulator activity scores reproduce the SSc-intrinsic subsets.

We analyzed the regulator activity scores in 4 independent publicly available SSc data sets from Milano et al. (7), Pendergrass et al. (6), Hinchcliff et al. (5), and Assassi et al. (14). For each data set, we first calculated sample-specific regulator activity scores using BASE (13) by integrating gene expression profiles and regulator target gene sets (see Methods). We applied intrinsic gene analysis to calculate a within-between score (15) to select regulators that showed the most consistent activity scores within an SSc-intrinsic subset but had the most variable activity scores across all subsets. The 270 regulators that had a FDR of less than 2% in at least 3 data sets were considered to be “intrinsic SSc regulators” and included activator proteins: CCAAT/enhancer-binding proteins; members of the E2F family, ETS family, STAT family, and GATA family; glucocorticoid receptors; interferon regulatory factors; RUNX1-related core binding factors; B cell– and T cell–related TFs; and numerous miRNAs (Supplemental Table 1; supplemental material available online with this article; https://doi.org/10.1172/jci.insight.137567DS1).

**Figure 2:**
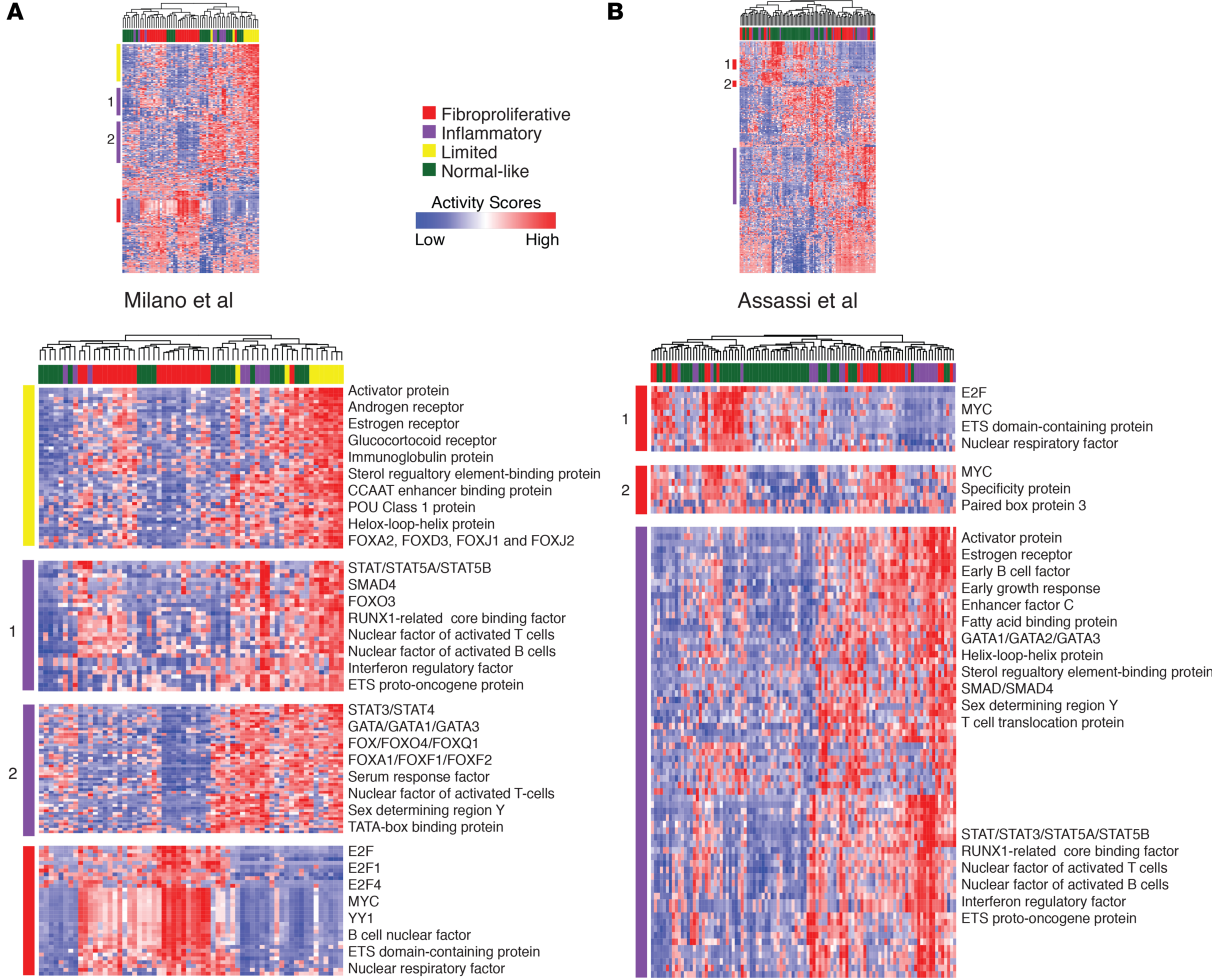
Activity scores reproduce intrinsic subtypes in SSc data sets. Heatmaps were plotted to show the intrinsic subsets clusters in the (**A**) Milano et al. (7) and (**B**) Assassi et al. (14) data sets. In the heatmap, each row represents a regulator, each column represents an SSc sample, and different colors infer intrinsic subtypes (red, fibroproliferative; purple, inflammatory; yellow, limited; and green, normal-like). The cells in the heatmap represent the normalized activity scores, within which blue denotes a low score and red denotes a high score. Driven regulators were listed for each cluster.

We organized the samples and regulators in each data set by hierarchical clustering of the activity scores with the 270 regulators. Broadly, we found that samples in the fibroproliferative subset clustered together and displayed activation of key regulators of cell proliferation, such as members of the E2F, MYC, and ETS families (Figure 2 and Supplemental Figures 1–4). Target genes of these fibroproliferative cluster regulators were highly enriched in cell cycle and DNA replication pathways (Supplemental Table 2 and Supplemental Figure 5; corrected *P* < 0.05), consistent with the activation of biological processes enriched in the fibroproliferative subset. Immune-related proteins, such as those from the STAT family, Runx1-related core-binding factors, and nuclear factor of activated T cells (NFAT), are enriched in the inflammatory subset (Figure 2 and Supplemental Figures 1–4). Their target genes are significantly involved in immune-related and signal transduction pathways, such as the B/T cell receptor signaling pathway, Th17 cell differentiation, and the TGF-β signaling pathway (Supplemental Table 2 and Supplemental Figure 6; corrected *P* < 0.05).

There was also a strong cluster of regulators for the Milano limited samples (7), which included 52 regulators, such as the glucocorticoid receptor, estrogen receptor, and androgen receptor (Figure 2A and Supplemental Figure 1). Surprisingly, in the Milano data set, although the limited and inflammatory subsets comprised different sets of regulators, the pathways to which these regulators mapped were highly shared (Jaccard score = 0.53; Supplemental Table 2). For instance, the MAPK signaling pathway, PI3K/Akt signaling pathway, and Wnt signaling pathway were all enriched in both clusters (Supplemental Table 2), suggesting that the pathways driving these subsets may be similar but that the regulators that activate the pathways are different. In the Assassi data set (14) (Figure 2B), 27 of 42 enriched pathways in the fibroproliferative samples were shared with those in the inflammatory samples and included the MAPK signaling pathway, the PI3K/Akt signaling pathway, and the Wnt signaling pathway (Supplemental Table 2). Similar results were found in the Pendergrass (6) and Hinchcliff data sets (5) (Supplemental Figures 3 and 4 and Supplemental Table 2).

### Regulator activity scores are correlated with MRSS.

**Figure 3:**
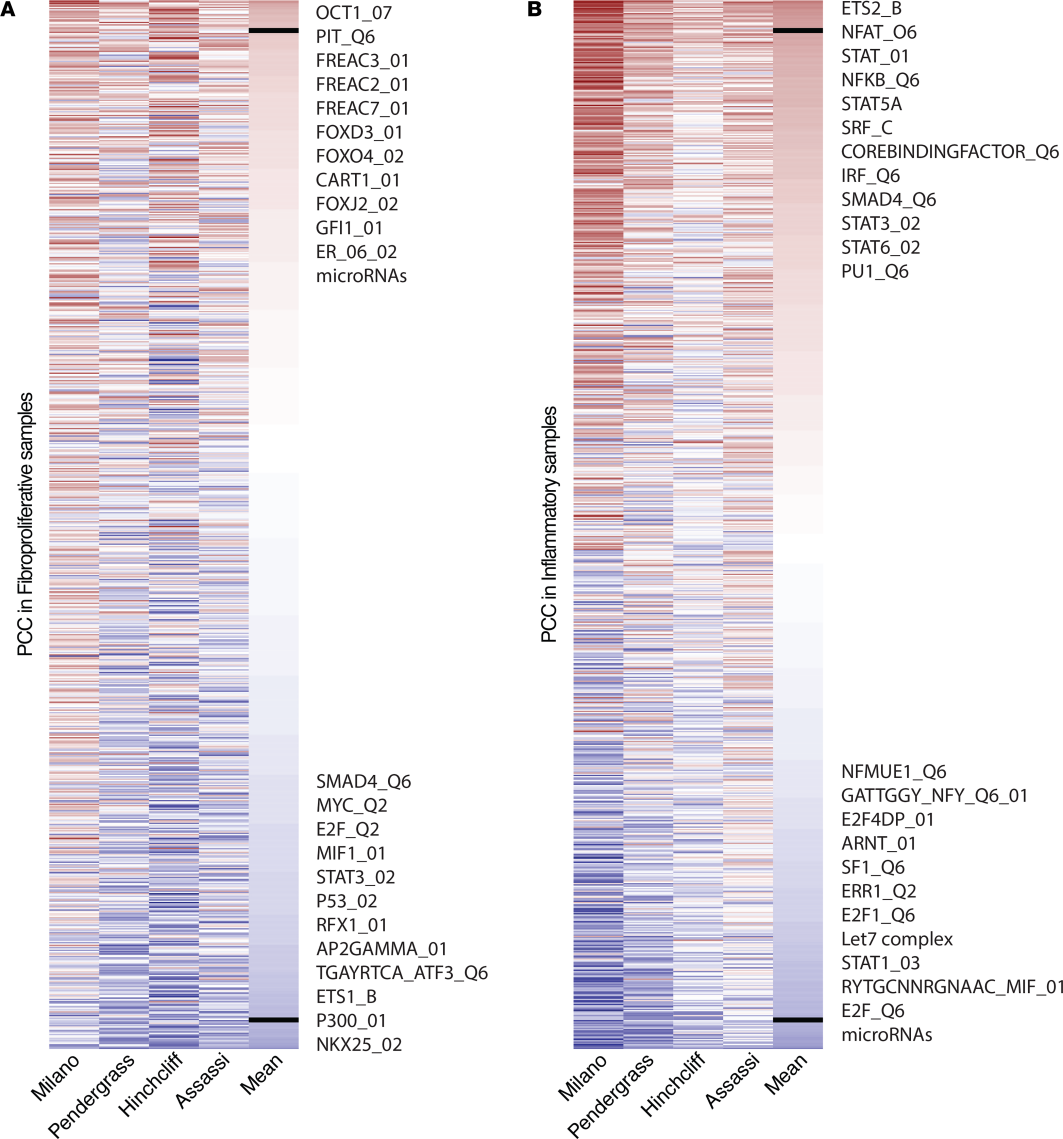
Identification of clinically relevant regulators. Heatmaps were plotted to show the Pearson correlation coefficients (PCC) between activity scores and the MRSS of samples across data sets for (**A**) fibroproliferative and (**B**) inflammatory SSc samples. In the heatmap, each row represents a regulator, each column represents a data set, and the cells in the heatmap represents PCC (within which blue is low PCC and red is high PCC). The median PCC was calculated for each regulator across data sets to show the correlation power. The heatmap was plotted by ranking the median PCC in decreasing order. The short, black, bold line is the cutoff we used to select out the common regulators in all data sets. Significant regulators are listed.

Next, we asked if regulator activity scores could be an additional index to identify patients with more severe disease. Correlations were calculated between each regulator’s activity score and patient MRSS in all 4 data sets. Mean correlations were calculated and ranked in decreasing order (Supplemental Tables 3 and 4 for fibroproliferative and inflammatory samples, respectively). We chose to focus on the 50 top- and bottom-ranked regulators. In the fibroproliferative subset, regulators that had the highest (correlation >0.17, Supplemental Table 5) and lowest (correlation <–0.21, Supplemental Table 6) correlation were identified. In particular, the activity scores of POU domain–related TFs (OCT1 and PIT1), Forkhead box (FOX) TFs, and many miRNAs were positively correlated with the MRSS of fibroproliferative SSc samples (Figure 3A). POU domain–related TFs function in the cell cycle regulation of immunoglobulins and are involved in viral infection (16). Regulators in the FOX family are well-known regulators of cell proliferation, e.g., FOXC1 increases the proliferation of fibroblast-like synoviocytes in autoimmune diseases (17). In contrast, activities of the tumor suppressor protein p53, ETS-related proteins, and immune-related TFs (including STAT3, SMAD4, and macrophage migration inhibitory factor) were negatively correlated with the MRSS (Figure 3A).

Similarly, for the inflammatory subset, as shown in Figure 3B, we identified the most and least correlated regulators, with the lowest positive correlation of 0.3 (Supplemental Table 7) and the highest negative correlation of –0.26 (Supplemental Table 8). Immune-related TFs, such as NFAT, nuclear factor κ light-chain enhancer of activated B cells (NF-κB), RUNX1-related core binding factor, STAT family, SRF, and interferon regulatory factor (IRF) were positively correlated with MRSS (Figure 3B). In contrast, we found that macrophage migration inhibitory factor, E2F family proteins, and several miRNAs were negatively correlated with the MRSS.

### Regulator interaction network in the context of SSc.

**Figure 4:**
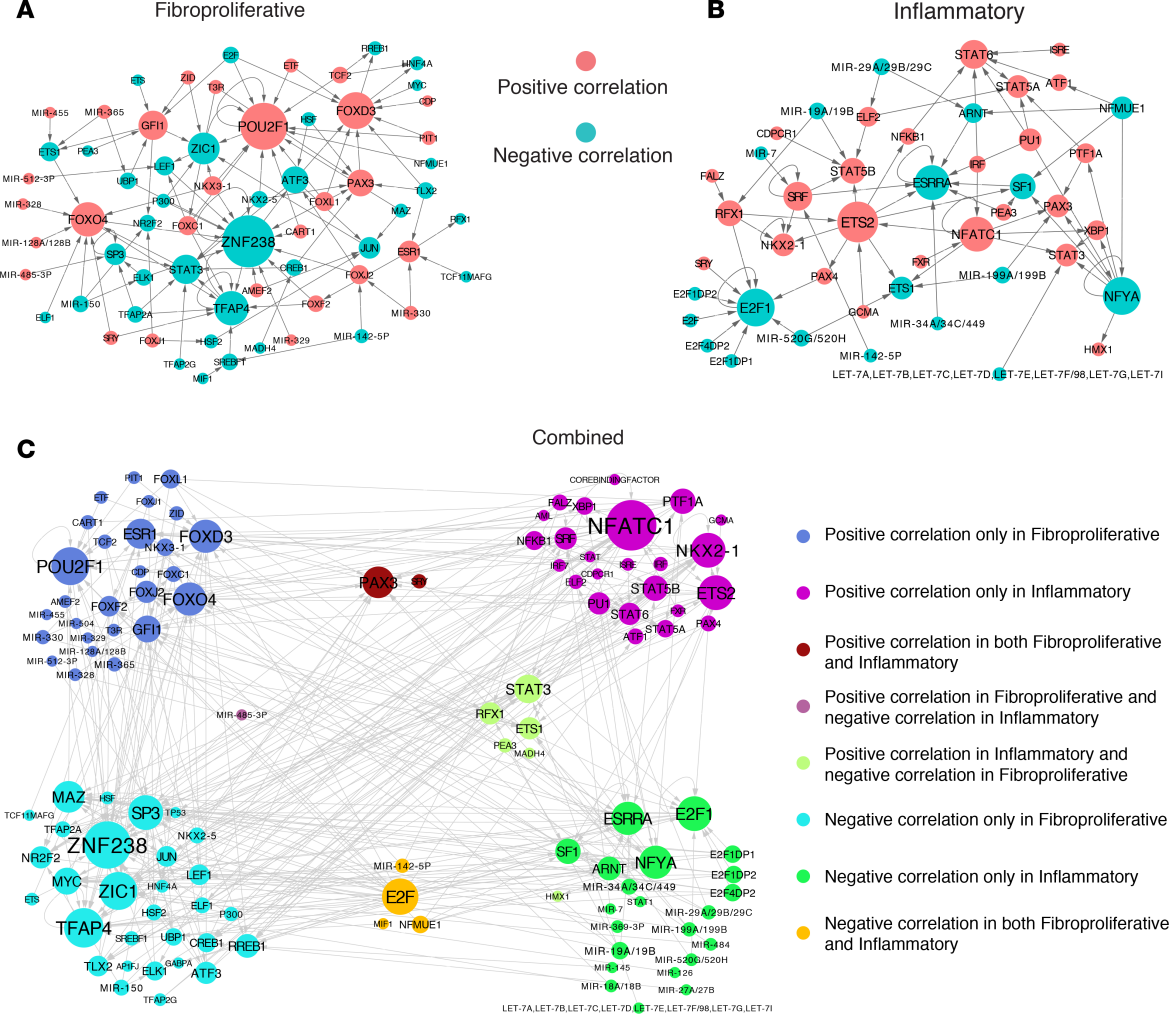
Regulator interaction networks in the context of SSc. Using the intrinsic subtype-specific clinically relevant regulators, we created networks in (**A**) only fibroproliferative samples; (**B**) only inflammatory samples; and (**C**) both of them. In networks (**A** and **B**), red nodes represent regulators whose activity scores are positively correlated with MRSS and cyan nodes represent regulators whose negatively scores are positively correlated with MRSS. The size of the node is positively correlated to its degree of connection. The arrow direction points from regulator to target and a circle denotes self-regulation. In the network of shown in **C**, different colors represent different clusters.

Based on the clinically relevant regulators that were identified, a network-based analysis was conducted to explore the interactions among those regulators in the context of SSc (Figure 1). We defined an interaction as established if a regulator’s target list contained another regulator listed in the MSigDB C3 database. We began by investigating the regulator-target interactions in the context of fibroproliferative (Figure 4A) and inflammatory (Figure 4B) samples. The 2 colors of nodes represent the type of correlation with the MRSS (red, positive; cyan, negative). The arrows indicate target directions, and a circular arrow indicates a self-regulating feedback loop. The size of a node indicates its relative degree of connection. Therefore, a larger node indicates that a given regulator plays a more important role by regulating others in SSc. For example, the TF SP3, which is negatively correlated with the MRSS of fibroproliferative samples, interacts with 5 other regulators, including cell-cycle–related regulators (miR-485-3p and TFAP2A) and immune-related regulators (STAT3 and ELK1) (Figure 4A). These results are in line with previous findings that SP3 is able to control IL-10 gene expression and interact with E2F1 (18, 19). Though SP1 is well studied in SSc, this suggests that, as a collagen metabolism regulator, SP3 may also play an important role in SSc etiology (20, 21).

Next, by combining the regulators associated with both fibroproliferative and inflammatory samples, we built a more comprehensive interaction network in the context of SSc (Figure 4C). Again, the size of a node indicates its degree of connection, and different colors of nodes represent different categories. Besides those regulators that have specific correlation directions with MRSS (4 corners in Figure 4C), we found that the activities of paired box 3 (PAX3) and sex-determining region Y (SRY), as shown in dark red, were positively correlated with MRSS in both fibroproliferative and inflammatory samples. PAX3 contributes to the embryonic development of the central nervous system and heart vasculature (22), which suggests that there are underlying associations between autoimmunity and the nervous system (23). Meanwhile, SRY has been shown to have a strong relationship with autoimmune diseases (24). MiR485-3p’s activities are positively and negatively correlated with the MRSS of fibroproliferative and inflammatory samples, respectively (purple node). Conversely, the activities of STAT3, ETS1, RFX1, PEA3, and MADH4 are positively and negatively correlated with the MRSS of inflammatory and fibroproliferative samples, respectively (light green nodes). Additionally, E2F, miR142-5p, MIF1, and NFMUE1 were negatively correlated with both intrinsic subsets of patients. Interestingly, we found that the activities of a subset of miRNAs were positively correlated with the MRSS in fibroproliferative samples and were negatively correlated with the MRSS in inflammatory samples.

### Regulator activity scores identify subgroups that differ by disease severity.

We then asked whether combinations of regulator pairs could identify novel subgroups of SSc within the intrinsic subsets that had more severe MRSS. We used a combination of gene expression data and clinical information collected from Milano et al. (7), Pendergrass et al. (6), and Hinchcliff et al. (5) to generate a larger cohort to increase the statistical power. The combined data from Franks et al. was used (25). Regulator activity scores and their correlation with the MRSS were recalculated based on the combined data. We again focused on the 50 top- and bottom-ranked regulators, which we reasoned were likely to be the most clinically relevant regulators in each intrinsic subset (Supplemental Table 9 for fibroproliferative and Supplemental Table 10 for inflammatory). Then, we performed pairwise analyses of regulators that were positively correlated with the MRSS in both fibroproliferative and inflammatory subsets.

**Figure 5:**
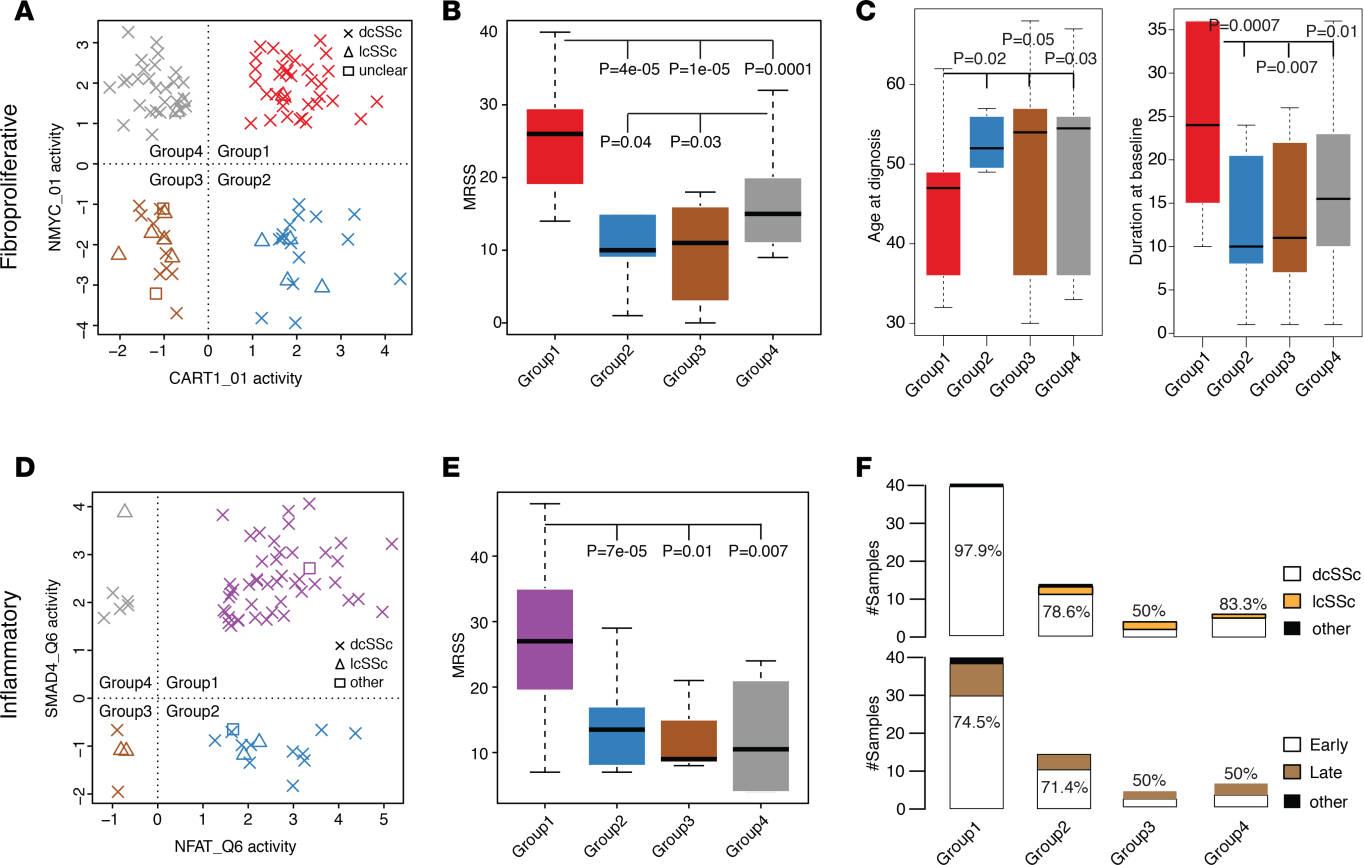
Regulator pairs identify subgroups of samples in intrinsic subsets. Shown are the sample distribution of (**A**) fibroproliferative and (**D**) inflammatory samples based on the activity scores of given regulator pairs. Group 1 (red dots for fibroproliferative and purple dots for inflammatory) samples have positive scores of both regulators. Group 3 (brown dots) samples have negative scores of both regulators. Group 2 (blue dots) and group 4 (gray dots) samples have 1 positive and 1 negative score. The box plot of MRSS comparisons between groups is shown in **B** for fibroproliferative and **E** for inflammatory samples. (**C**) Box plots for age at diagnosis and disease duration at baseline are shown for each group of fibroproliferative samples. Two-tailed Mann-Whitney-Wilcoxon test *P* values are listed. (**F**) Clinical subtypes and stage fractions for each group are shown in inflammatory samples. Fractions of dcSSc and early-stage samples are shown.

For the pairwise combinations of regulator activity scores in the fibroproliferative subset, a total of 1225 pairs were divided into 4 subgroups, using 0 as cutoff for each activity score. By comparing MRSS between groups, we found 910 pairs where samples that had positive activity of both regulators (i.e., double positive) also had more severe skin disease compared with those in other 3 groups (FDR <1%, Supplemental Table 11). For example, a top-ranked pair, CART1_01 and NMYC_01, classified fibroproliferative samples into 4 subgroups: those that were positive for both regulators (red, group 1); those positive for CART1_01 and negative for NMYC_01 (blue, group 2); those that were negative for both regulators (brown, group 3); and those that were negative for CART1_01 and positive for NMYC_01 (gray, group 4), as shown in Figure 5A. CART1, known as TNF receptor–associated factor 4, is able to target fibroblasts (26) and regulate cell-cycle pathways (27, 28). NMYC, a member of the MYC family, plays an important role in regulating cell cycle and metabolism in many human diseases (29). Similar observations were found among the significant regulator pairs, suggesting that a potentially large number of regulators are dysregulated in SSc (Supplemental Table 11).

We found that patients with samples in group 1 had more severe disease than patients with samples in the other groups (all 2-sided Wilcoxon rank test; *P* < 0.0005; Figure 5B). The fraction of samples from patients with dcSSc in group 1 was similar to that in group 4 (Supplemental Figure 7), but a significant difference in MRSS persisted. The fraction of samples from patients with dcSSc in group 1 was 1.23-fold and 1.65-fold higher than groups 2 and 3, respectively (2-tailed Fisher’s exact test; *P* = 0.04 and *P* = 0.0008). Patients with samples in group 4 had higher MRSS compared with patients with samples in groups 2 and 3 (all 2-sided Wilcoxon rank test; *P* < 0.05; Figure 5B), likely due to the higher prevalence of dcSSc. These observations suggest that patients with higher NMYC activity are more likely to have dcSSc and that patients with higher NMYC activity have a worse disease phenotype than those that have lower NMYC activity. Patients with samples in groups 1 and 2 had relatively higher fractions of early-stage disease than those in groups 3 and 4, which might suggest that higher CART1 activity is associated with early-stage disease (Supplemental Figure 7). Moreover, we found that samples in group 1 came from individuals who were significantly younger at diagnosis and had notably longer disease durations at baseline compared with other groups (all 2-sided Wilcoxon rank test; *P* ≤ 0.05; Figure 5C). We stratified patients by treatment to determine if the subgroups could derive from treatment with disease-modifying antirheumatic drugs. We found that samples in group 1, 3, and 4 came primarily from individuals who were not on immunosuppressive treatment at baseline (Supplemental Table 12). A slightly larger proportion of baseline samples from group 2 had mycophenolate mofetil treatment (47.4%) compared with those who had no treatments (31.6%). Therefore, treatment does not appear to be a defining feature of the groups.

We repeated the pairwise analyses for patients in the inflammatory subset and identified 312 pairs where patients that were double positive had a more severe disease phenotype (Supplemental Table 13). For example, using a top-ranked combination of NFAT (NFAT_Q6) and SMAD4 (SMAD4_Q6), samples from patients were divided into positive/positive (group 1, purple), positive/negative (group 2, blue), negative/negative (group 3, brown), and negative/positive (group 4, gray) groups (Figure 5D). NFAT is a well-known TF that has a significant role in the immune system (30). SMAD4 plays important roles in the TGF-β and fibroblast growth factor signaling pathways (31). Again, patients with samples in group 1 had significantly higher MRSS than those in other groups (all 2-sided Wilcoxon rank test; *P* < 0.05; Figure 5E). Similar to our results with the fibroproliferative subset, we found that samples in group 1 were more likely to be from patients with dcSSc compared with other groups (Figure 5F, 1.25-fold and 1.96-fold difference when comparing dcSSc fraction in group 1 to that in groups 2 and 3, respectively; 2-tailed Fisher’s exact test; *P* = 0.03 and *P* = 0.01). Group 1 samples had a relatively higher fraction of samples coming from patients with dcSSc than in group 4 (1.18-fold). Interestingly, this observation was found in many immune system–related regulator pairs as well (Supplemental Table 12). We found that group 1 and group 2 samples were more likely to be from early-stage patients (mean disease duration of 27.15 and 24 months since first onset of non-Raynaud’s symptoms, respectively), compared with group 3 (mean disease duration of 106 months) and 4 (disease duration of 33.83 months) (Figure 5F). Unlike the fibroproliferative subset, we did not find significant differences between the age and disease duration among patients grouped using these subgroups among the inflammatory subset. We also examined treatment effects on these subgroups. We found that the majority of samples in group 1 and 2 were from individuals that were not on immunosuppressive therapies (61.7% and 62.5%, respectively; Supplemental Table 14). Samples in group 3 and 4 came from individuals for which 50% and 44%, respectively, were not on an immunosuppressive therapy.

### Subgroups associate with FVC decline, a surrogate for interstitial lung disease.

**Figure 6:**
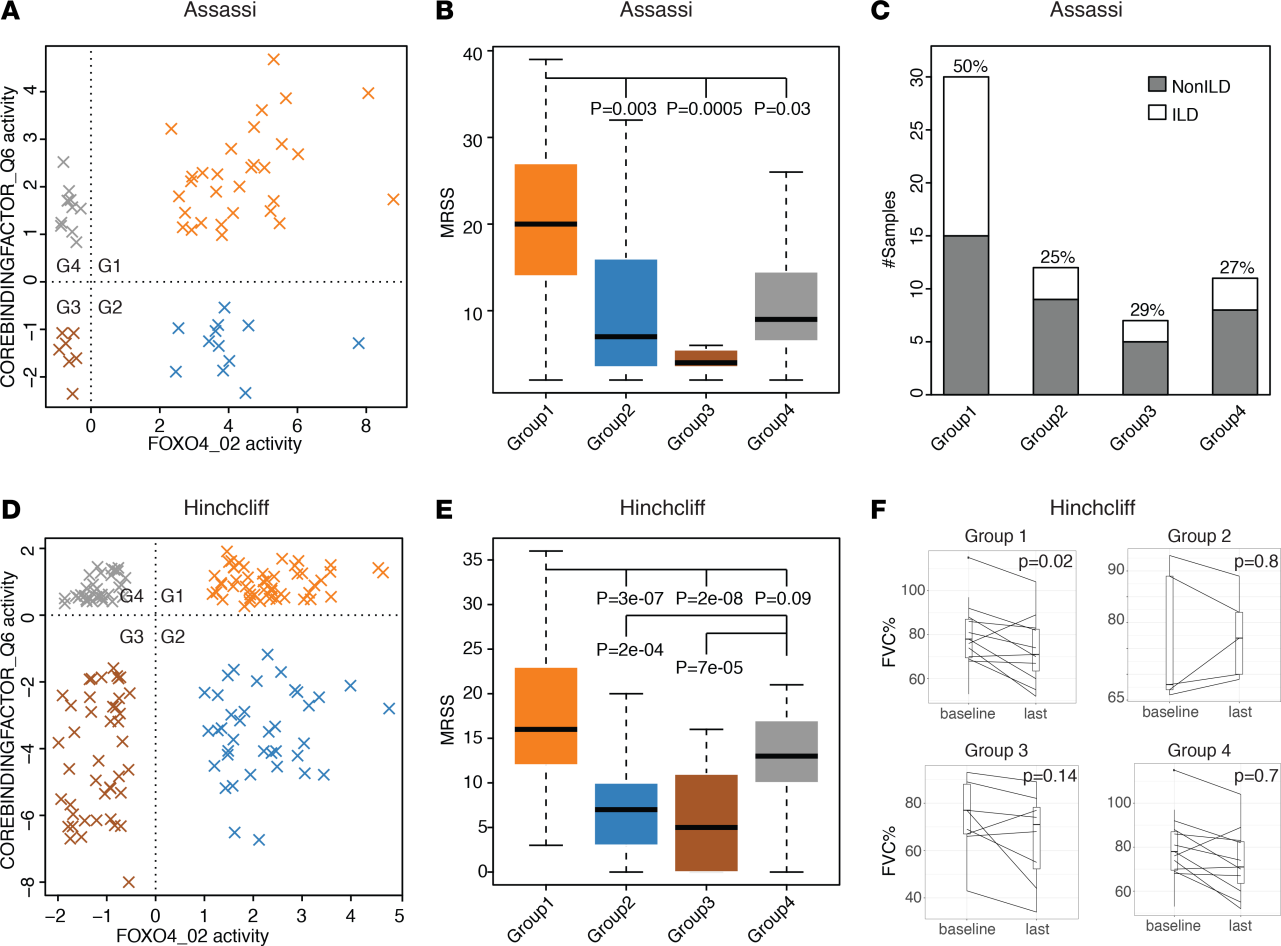
Regulator pairs associated with SSc complicated by pulmonary fibrosis. (**A**) The distribution of samples between ILD and non-ILD SSc samples. (**B**) MRSS comparisons. (**C**) Bar plot of the fraction of ILD samples in each group from the Assassi data set (14) with a given regulator pair. (**D**) Validation of sample distribution. (**E**) MRSS comparisons from the Hinchcliff data set with the same regulator pair (5). (**F**) Validation with patients using their adjusted forced vital capacity (FVC).

Leveraging the patients with interstitial lung disease (ILD) and without ILD contained within the Assassi data set (14), we determined if novel subgroups were associated with the presence of ILD (FVC reduction). By performing the pairwise analyses of the common regulators across 4 data sets in both fibroproliferative and inflammatory subsets, we classified 23 patients with SSc with ILD and 37 patients with SSc without ILD into 4 subgroups, where group 1 patients had the worst disease phenotype compared with other groups (FDR <5%). Additionally, for each single pair, we calculated the fold change of the fraction of ILD between group 1 and other groups. As a result, we identified 28 pairs of regulators with a fold change greater than 1.5 (Supplemental Table 15). For example, using the top-ranked pair of FOXO4_02 and COREBINDINGFACTOR_Q6, patients were classified into 4 groups, as shown in Figure 6A. FOXO4 is known to decrease the activity of hypoxia-inducible factor, which is a validated biomarker for lung diseases (32–34). The core binding factor, which is within the RUNX family, has been shown to be highly involved in autoimmunity and inflammation and several autoimmune diseases and contributes to pulmonary fibrosis (35–37). Again, patients in group 1 had the highest MRSS compared with that in other groups (all 2-sided Wilcoxon rank test; *P* < 0.05; Figure 6B). The majority of patients in each group were not on immunosuppressive therapies (Supplemental Table 16). We also found that patients in groups 1 and 4 were more likely to have dcSSc than those in groups 2 and 3 (Supplemental Table 14). In contrast, patients in groups 2 and 3 were more likely to have lcSSc than those in groups 1 and 4. This suggests that the activity of the core binding factor might be higher in patients with dcSSc versus lcSSc. More patients in group 1 had ILD (50%) compared with other groups (25%–29% ILD; Figure 6C), which is consistent with a previous study showing that patients with dcSSc are more likely to develop fibrotic pulmonary complications (1). Patients in groups 2 and 3 were more likely to be classified in the normal-like intrinsic subset compared with those in the rest of the groups (Supplemental Table 16).

We repeated the analysis in the skin biopsy samples analyzed in Hinchcliff et al. (5), as shown in Figure 6D. Patients with unclear ILD diagnosis at baseline or with morphea were excluded. We found that group 1 samples were from patients with more severe skin involvement than those in the other 3 groups (Figure 6E). Again, the majority of samples in group 1 (85.7%) and group 4 (82.7%) were from patients with dcSSc compared with other groups (Supplemental Table 17); samples in groups 2 and 3 were more likely to be from normal-like patients when compared with the other groups. We did not find enrichment of ILD in group 1 at initial biopsy but did find those patients had an increased rate of FVC decline over 36 months. ILD was present in group 1 (45%), group 2 (67%), group 3 (70%) and group 4 (45%) (Supplemental Figure 8).

We hypothesized that the observed stratification might result from the majority of patients in the Hinchcliff data (5) set being early stage (66.7% samples from patients in group1 had SSc disease duration <18 months at baseline), while only 2 patients in the Assassi data set (14) were early stage. To test this, patients were stratified into groups based on their baseline biopsies. FVC predicted values were compared between baseline and the last time point (>36 months). We found that patients in group 1 had a significant FVC decline compared with other groups (2-tailed paired samples *t* test, *P* = 0.02, Figure 6F) that was not observed in the other groups.

SScMH_06 was the only patient that lacked ILD at baseline but had developed ILD by the last time point. This patient’s baseline biopsy was classified in group 1. Analysis of patient’s pulmonary function measures showed that FVC, first second of forced expiration (FEV1) and adjusted diffusing capacity of carbon monoxide (DLCOadj) decreased over time (Supplemental Figure 8). These observations suggest that using the combination of core binding factor and FOXO4 captures the features of FVC decline in SSc skin biopsies.

To further validate this finding, we applied an activity score calculation to an independent PBMC data set (Cheadle et al., ref. 38). Using the same pair of regulators, we again found that samples were able to be divided into 4 groups (Supplemental Figure 9). Notably, half of the patients in group 1 had ILD, which is the largest ILD fraction compared with other groups (16.7%–33.3% ILD, Supplemental Figure 9). These observations suggest that the use of regulator pairs, such as core binding factor and FOXO4, enables identification of a subgroup of patients with SSc that have worse skin disease and are more likely to have ILD. These findings indicate that our skin regulator signature is also applicable to blood samples.

## Discussion

In order to understand the functions of regulators of gene expression in the context of SSc, our computational framework used gene expression profiles and target gene lists of regulators to calculate the activity scores from mRNA expression data. Our results are robust because we included 5 independent SSc gene expression data sets (Table 1).

Our results demonstrate that intrinsic subsets were clustered together based on regulator activity scores (Supplemental Figures 1–4). Interestingly, we found that a small number of patients with SSc identified as “normal-like” by gene expression grouped with inflammatory or fibroproliferative subset when ordered using regulator activity. This may have been the result of using all genes in the genome that passed basic quality filters to infer regulator activity score rather than a more limited intrinsic gene list. Activity scores could present a future opportunity to further investigate patients classified as “normal-like” by gene expression.

The intrinsic subset classification system has been used to stratify patients in SSc clinical trials (25) and may predict response to therapy (39–41). The TF signatures reported here may assist in further stratification of patients with SSc within the existing intrinsic subsets in both cross-sectional and longitudinal studies. We hope the data we generate here will provide mechanistic insight into these subsets for personalized medicine in SSc, as they provide a comprehensive view of the regulators that underlie the intrinsic gene expression subsets, and provide the opportunity to mechanistically understand the regulatory networks that give rise to these different groups of patients.

An intriguing result from this analysis was that although each intrinsic subset often contained different regulators, the enriched pathways that were activated showed a high level of consistency (Supplemental Table 2). For example, we found the MAPK signaling, P13K/Akt signaling, and TGF-β signaling pathways were consistently identified across subsets, which suggest they are essential pathways for SSc pathogenesis. Four KEGG pathways, chronic myeloid leukemia, endocrine resistance, the longevity regulating pathway, and transcriptional misregulation in cancer were all shared by fibroproliferative, inflammatory, and limited samples in the Milano data set (7) (the remaining 3 data sets lacked limited patients with SSc). Several microbiome-related pathways were enriched in inflammatory subsets, suggesting an important relationship between SSc and microbiome (Supplemental Table 2) (42, 43).

The results of these analyses provide a catalog of TFs and miRNAs that are deregulated in SSc and provide information about their correlation with clinical covariates associated with skin and lung disease. We hope this information can now be used to study the mechanisms of SSc in these patients with more severe disease. There were a number of immune-related regulators that were associated with more severe disease (Figure 4). A regulator’s correlation with MRSS across data sets provides one method by which regulators could be prioritized for further study (Supplemental Tables 5–8). As an example, the activity of FOX family TFs was positively correlated with MRSS in the fibroproliferative subsets, while E2F family regulators were negatively correlated with MRSS in the fibroproliferative and, surprisingly, also in the inflammatory subsets (Figure 4C). Previous studies have shown that a dysfunction in E2F signaling enhances the function of inflammatory cytokines and leads to autoimmunity (44–46).

We repeatedly identified the RUNX1-related core binding factor to be a regulator of the inflammatory subset (Figures 2–4). Patients with ILD with higher RUNX1-related core binding factor activities were more likely to be dcSSc and were more likely to have inflammatory signatures (Supplemental Table 16). In contrast, samples from patients with lower RUNX1-related core binding activities were more likely to be from normal-like patients (Supplemental Table 16). Additionally, we found that high expression of 2 other RUNX1-related core binding factors (AML1_01 and AML1_Q6) identified inflammatory subsets with the highest MRSS (mean PCC = 0.28). RUNX1-related core binding factors are essential immune regulators (36, 37), and decreased expression has been reported in SSc Tregs (47). Our regulator interaction network in inflammatory subsets suggests RUNX1 may be a central regulator of inflammatory processes in SSc end target tissues (Supplemental Figure 10).

We also identified potentially novel subgroups within fibroproliferative and inflammatory subsets that may be at risk for more severe disease (Figure 5). Samples from patients assigned to a double-high regulator group were more likely to be from patients have higher MRSS and with dcSSc. Analysis of multiple data sets suggests that patients in these double-high groups were more likely to have FVC decline. We first identified this in Assassi et al. (14) and validated it in independent skin and PBMC data sets, which suggests that the regulator pairing is informative across multiple tissues. The results suggested that disease-modifying antirheumatic drugs were not a major confounder or driver of these novel subgroups (Supplemental Tables 12, 14, and 16).

Limitations of our analyses include the small number of samples of limited intrinsic subset across the cohorts and the dependence on published clinical data. The Milano (7) and Hinchcliff (5) data sets contained small numbers of limited patients (*n* = 7 and *n* = 2, respectively). There were no patient samples in Pendergrass (6) or Assassi (14) data sets classified as limited. Although, we did observe a cluster of TFs for the limited patients in Milano (7), and we have listed the enriched pathways in Supplemental Table 2, it was not possible to validate these results in an independent data set. A second limitation is that our definitions of ILD were variable across cohorts. In these analyses, ILD was defined as in each of the original publications with the exception of the Hinchcliff cohort (5), where ILD was defined using the criteria defined in the methods.

In summary, we applied computational approaches to investigate the function of regulators associated with SSc pathogenesis. Though SSc is a complex and heterogeneous disease, taking advantage of the previously well-defined intrinsic subsets, we identified the most significant and highly correlated TFs for fibroproliferative and inflammatory patients. These observations might provide a list of novel regulators for those distinct patients with SSc. The prediction of ILD could provide additional information to determine probability of ILD development over time.

## Methods

### Data set collection.

Two types of data sets were used, gene expression data sets from patients with SSc and publicly available target gene lists for regulators. The regulator target gene lists and motif gene sets (C3) were downloaded from MSigDB (https://www.gsea-msigdb.org/gsea/msigdb/index.jsp) (48, 49) (November 2017), which totaled 836 gene sets, of which 615 were TFs and 221 were miRNAs. Five independent SSc gene expression data sets were obtained from the gene expression omnibus (GEO) (50), as shown in Table 1. Milano (GSE9285) (7), Pendergrass (GSE32413) (6), and Hinchcliff data sets (GSE59787) (5) were generated by analysis of skin biopsies on Agilent Technologies 2-channel DNA microarrays in a common reference design and included 75, 89, and 165 samples, respectively. For these 3 data sets, both lesional (forearm) and nonlesional (lower back) skin biopsies were collected and processed using similar protocols, as described in the original studies. These studies have shown the consistent gene expression between lesional (forearm) and nonlesional (lower back) samples. Intrinsic subset assignments were as defined in the original publications. Assassi (GSE58095) (14), contains gene expression data collected from 102 SSc involved forearm skin biopsies using a single-channel DNA microarray, and intrinsic subset assignments were determined in a separate study using a previously trained classifier (25). Additionally, we downloaded an independent PBMC RNA gene expression data set, Cheadle et al. (GSE33463) (38), that contains blood from 27 patients with SSc, 8 of whom had ILD as previously defined. Clinical data, such as MRSS, disease duration, and ILD status for each data set were either obtained from NCBI GEO or were requested from the authors of each study. Early SSc was defined using criteria for each published data set (usually as SSc disease duration less than 18 months from the time of the first non-Raynaud’s symptom attributed to SSc to the sample collection time).

### SSc-ILD definition across data sets.

In this meta-analysis of published cohorts, presence or absence of ILD used was as defined in each of the original publications. These were as follows. For the Assassi et al. data set (14), which was used as the training data set, patients were classified as having ILD when the percentage FVC predicted was less than 70%. For the Hinchcliff et al. data set (5), which was used as validation cohort, ILD was defined as the presence of radiographic findings consistent with ILD in the opinion of an expert thoracic radiologist (5, 51). The Cheadle et al. data set (38), also used as a validation cohort, defined ILD according to pulmonary function tests and chest high-resolution computed tomography.

### Regulator activity score calculation.

To implement our computational pipeline, we first imputed missing gene expression values using the mean value of a probe across samples. Then, probe-level DNA microarray data were collapsed to genes using median values. Next, by integrating target genes and gene expression profiles, we applied a statistical algorithm called BASE (13) to calculate a regulator activity score for each regulator in each sample. This algorithm has been successfully applied in tumors to infer the activity of regulators based on target gene expression (52, 53). By quantile normalizing the gene expression profile, BASE ensures that samples have a comparable distribution at the genetic level. Then, for single-channel DNA microarray data sets, BASE normalizes its gene expression based on the median expression of each gene. For 2-channel DNA microarray data, as the data has already been processed by calculating log ratios, no additional processing is needed. Afterward, for a regulator whose target gene list is *g* = {*g*_1_...,*g_i_*...,*g_n_*} (if gene_i_ is target gene, *g_i_* is 1; otherwise *g_i_* is 0) and a sample with gene expression profile is *e* = {*e*_1_...,*e_i_*...,*e_n_*}, where *n* is the number of genes, BASE sorts the gene expression in decreasing order and generates 2 cumulative distribution functions. The first function calls the foreground function, which captures the gene expression levels of target genes of this regulator. Then, the background function is calculated to represent the gene expression levels of nontarget genes. When target genes have a higher gene expression level, the foreground function increases dramatically and the background function increases slowly. When the target genes have lower levels of gene expression, the foreground function increases slowly and the background function increases dramatically. The maximum difference of these 2 functions was used as a preliminary regulator activity score in this sample. A higher score indicates that the target genes of a given regulator are being more highly expressed, which translates to a high regulator activity. Further, by performing a permutation that randomly permutes *g* for 1000 times, BASE recalculates the regulator activity score to provide a score vector *s_p_* = {*s*_1_,*s*_2_...,*s*_1000_}. Finally, by dividing the mean of absolute values of *s_p_*, BASE normalizes the preliminary regulator activity score and infers a sample-specific regulatory activity score. As a general approach, we considered TF activity scores as being positively correlated and miRNA activity scores as being negatively correlated with the expression of their target genes. We recognize that there are cases that violate these assumptions (i.e., transcriptional repressors and activating miRNAs) that will have to be considered on an individual gene basis. Z-transformed activity scores for each data set are listed in Supplemental Table 18.

### Common regulator identification.

Using the calculated regulatory activity scores, we calculated the correlations between scores and the MRSS that were obtained at the time of the skin biopsy. Mean correlation was calculated across all cohorts and ranked in decreasing order. The 50 most top- and bottom-ranked regulators were considered to be clinically relevant regulators.

### Network construction.

To build the regulator interaction network, we applied our previous workflow (53). After mapping regulators to the genetic level, an interaction could be identified in the case when the target gene list of a regulator contains the genetic symbol of other regulators within the MSigDB C3 database. To reduce the complexity of the network, duplicated regulators were amalgamated, and unclassified motifs were excluded. In the network, different colors implied the correlation directions (positively or negatively) with MRSS. The arrow on the edges showed the regulatory direction and a circle with an arrow indicates that a regulator participates in a self-feedback loop. The network was created using Cytoscape (54).

### Statistics.

We calculated within-between scores for each regulator in each data set using the intrinsic subsets as groups (15). FDR was provided to define the degree of consistent activity in 1 intrinsic subset and the degree of the diverse activity between intrinsic subsets. An FDR of 2% was used as a statistical significance cutoff, as the results showed the most consistency with the previously defined intrinsic subsets.

Pathway enrichment analysis was conducted via g:Profiler (55) with best per-parent group setting. The KEGG pathway data set was selected (56). A corrected *P* value of less than 0.05 for multiple testing using the default g:SCS method was applied to define the significantly enriched pathways.

For the subgroup analysis, *P* values between groups were calculated by 2-tailed Mann-Whitney-Wilcoxon test. Then, the FDR was calculated with the p.adjust() function in R. For subgroups in intrinsic subsets, an FDR of <1% was used to define significance. Given their smaller sample size, an FDR of <5% was used to define significance for subgroups in ILD samples. For the FVC comparisons between baseline and the last time point, 2-tailed paired-samples *t* test was used.

Heatmaps were plotted using the heatmap.2() function in R “gplots” package (57). The activity scores were processed with the scale() function in R.

## Author contributions

YW and MLW designed the study. YW, JMF, DMT, and TAW collected the data. YW performed the research and analyzed the data. JMF determined intrinsic subsets. MH and MY collected the data for pulmonary fibrosis. YW and DMT performed the analyses of pulmonary fibrosis. YW and MLW wrote the manuscript. All authors discussed the results and have read and edited the manuscript.

## Supplementary Material

Supplemental data

Supplemental Table 1

Supplemental Table 2

Supplemental Table 3

Supplemental Table 4

Supplemental Table 5

Supplemental Table 6

Supplemental Table 7

Supplemental Table 8

Supplemental Table 9

Supplemental Table 10

Supplemental Table 11

Supplemental Table 12

Supplemental Table 13

Supplemental Table 14

Supplemental Table 15

Supplemental Table 16

Supplemental Table 17

Supplemental Table 19

## Figures and Tables

**Table 1:**
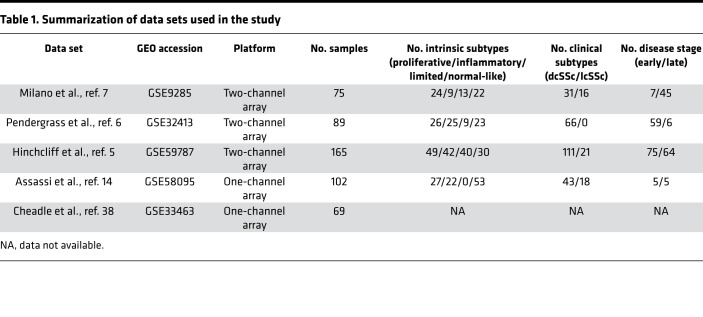
Summarization of data sets used in the study

## Version 1 07/28/2020

In-Press Preview

## Version 2 09/03/2020

Electronic publication
