# Supplementary material for: Regulator combinations identify systemic sclerosis patients with more severe disease
Source: JCI Insight. 2020 Sep 3;5(17):e137567. doi: 10.1172/jci.insight.137567 (PMC7526449; doi:10.1172/jci.insight.137567)

Supplemental Figures

Supplemental Figure S1. **Activity heatmap of intrinsic subsets related regulators in Milano dataset.** Each row is a SSc sample where red infers fibroproliferative subset, purple infers inflammatory subset, yellow infers limited subset, light green infers normal-like sample and dark green infers healthy control. Each cell is the activity score of one regulator (column) in one sample (row). Blue and red cells mean low and high activities, respectively. Intrinsic subset specific regulator clusters are highlighted with black boxes and names. The name works in pathway enrichment analyses (supplemental table S2).

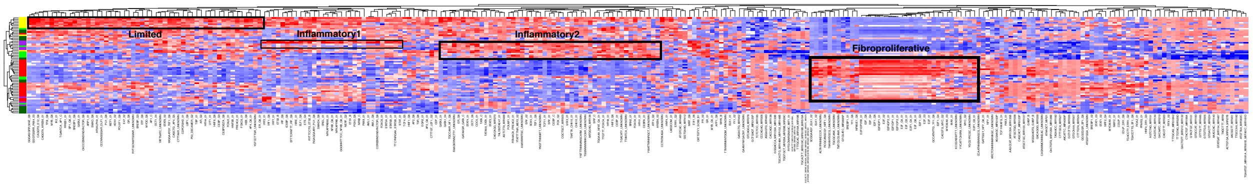

Supplemental Figure S2. **Activity heatmap of intrinsic subsets related regulators in Assassi dataset.** Each row is a SSc sample where red infers fibroproliferative subset, purple infers inflammatory subset, light green infers normal-like sample and dark green infers healthy control. No limited intrinsic subset in Assassi dataset. Each cell is the activity score of one regulator (column) in one sample (row). Blue and red cells mean low and high activities, respectively. Intrinsic subset specific regulator clusters are highlighted with black boxes and names. The name works in pathway enrichment analyses (supplemental table S2).

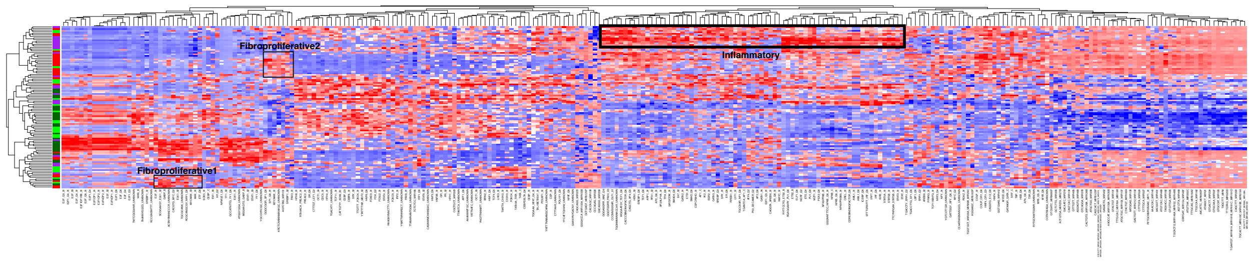

Supplemental Figure S3. **Activity heatmap of intrinsic subsets related regulators in Pendergrass dataset.** Each row is a SSc sample where red infers fibroproliferative subset, purple infers inflammatory subset, light green infers normal-like sample and dark green infers healthy control. No limited intrinsic subset in Pendergrass dataset. Each cell is the activity score of one regulator (column) in one sample (row). Blue and red cells mean low and high activities, respectively. Intrinsic subset specific regulator clusters are highlighted with black boxes and names. The name works in pathway enrichment analyses (supplemental table S2).

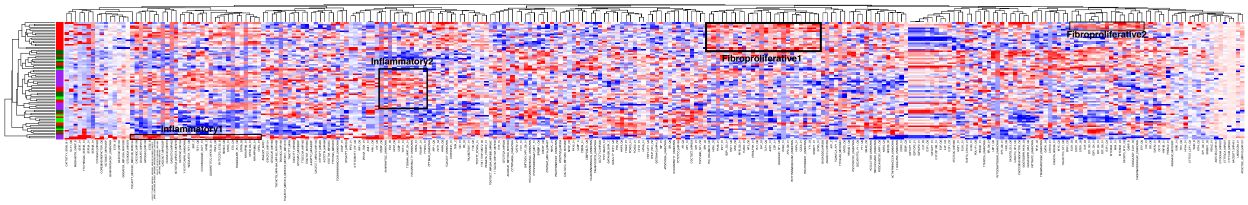

Supplemental Figure S4. **Activity heatmap of intrinsic subsets related regulators in Hinchcliff dataset using baseline samples.** Each row is a SSc sample where red infers fibroproliferative subset, purple infers inflammatory subset, light green infers normal-like sample and dark green infers healthy control. No limited intrinsic subset in Hinchcliff dataset. Each cell is the activity score of one regulator (column) in one sample (row). Blue and red cells mean low and high activities, respectively. Intrinsic subset specific regulator clusters are highlighted with black boxes and names. The name works in pathway enrichment analyses (supplemental table S2).

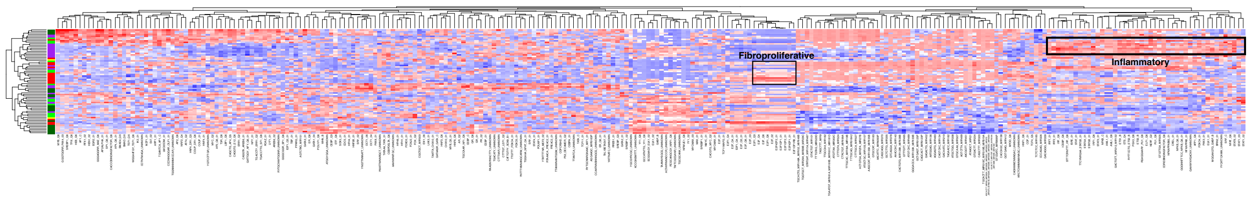

Supplemental Figure S5. **Common pathways enriched in fibroproliferative samples across four datasets.** Pathway details are shown in supplemental table S2. Common pathways are listed.

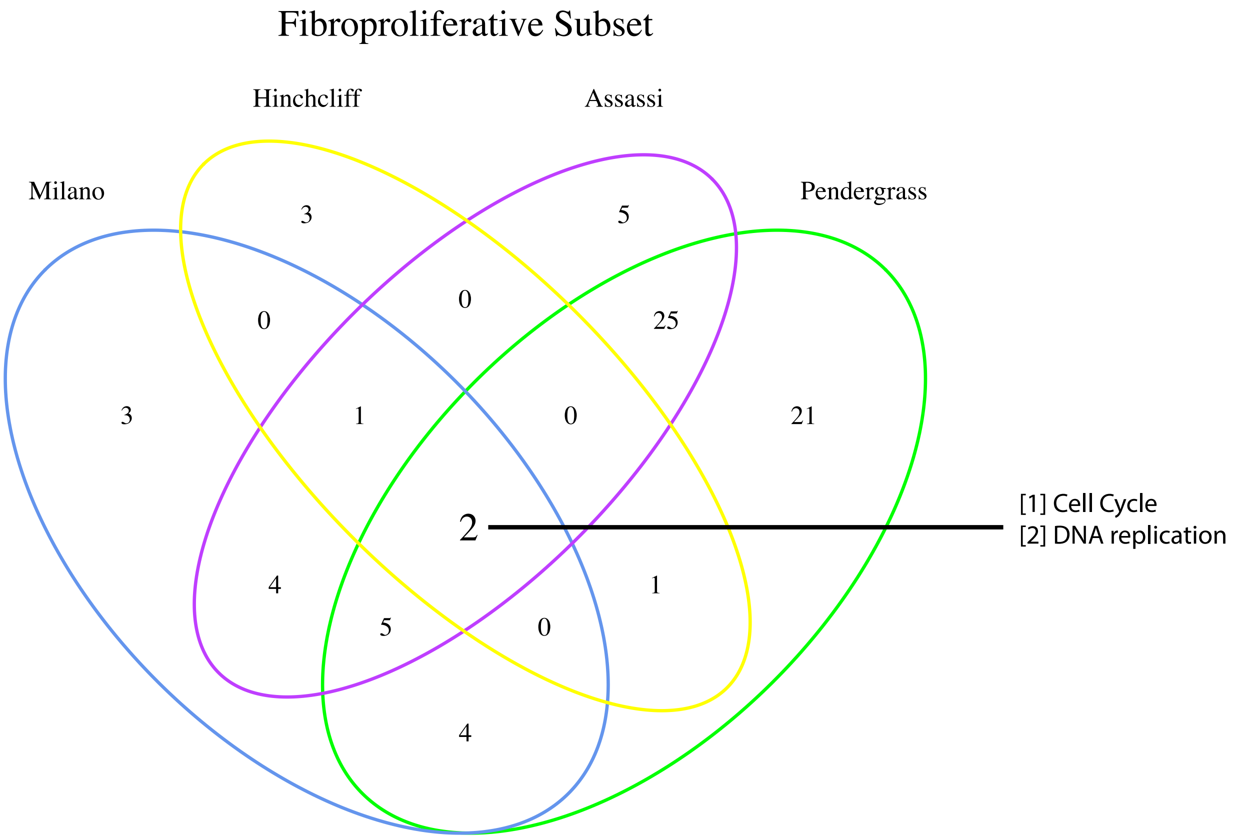

Supplemental Figure S6. **Common pathways enriched in inflammatory samples across four datasets.** Pathway details are shown in supplemental table S2. Common pathways are listed.

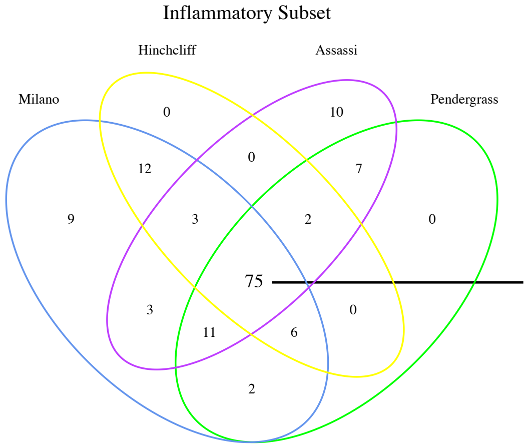

[1] Pathways in cancer
[2] Transcriptional misregulation in cancer
[3] PI3K-Akt signaling pathway
[4] C-type lectin receptor signaling pathway
[5] Focal adhesion
[6] MAPK signaling pathway
[7] Ras signaling pathway
[8] TNF signaling pathway
[9] Proteoglycans in cancer
[10] Chemokine signaling pathway
[11] T cell receptor signaling pathway
[12] Cytokine-cytokine receptor interaction
[13] Rap1 signaling pathway
[14] Th1 and Th2 cell differentiation
[15] Human T-cell leukemia virus 1 infection
[16] B cell receptor signaling pathway
[17] JAK-STAT signaling pathway
[18] Chronic myeloid leukemia
[19] Human papillomavirus infection
[20] Breast cancer
[21] Axon guidance
[22] Osteoclast differentiation
[23] Prostate cancer
[24] Regulation of actin cytoskeleton
[25] Th17 cell differentiation
[26] Cellular senescence
[27] Melanoma
[28] ErbB signaling pathway
[29] Small cell lung cancer
[30] Endocrine resistance
[31] Inflammatory bowel disease (IBD)
[32] Gastric cancer
[33] Human cytomegalovirus infection
[34] Hepatitis B
[35] EGFR tyrosine kinase inhibitor resistance
[36] AGE-RAGE signaling pathway in diabetic complications
[37] Neurotrophin signaling pathway
[38] Wnt signaling pathway
[39] Acute myeloid leukemia
[40] Non-small cell lung cancer
[41] Signaling pathways regulating pluripotency of stem cells
[42] Fc epsilon RI signaling pathway
[43] Chagas disease (American trypanosomiasis)
[44] Glioma
[45] FoxO signaling pathway
[46] Parathyroid hormone synthesis, secretion and action
[47] Pancreatic cancer
[48] Thyroid hormone signaling pathway
[49] Relaxin signaling pathway
[50] Shigellosis
[51] Colorectal cancer
[52] HIF-1 signaling pathway
[53] Platelet activation
[54] Fc gamma R-mediated phagocytosis
[55] Longevity regulating pathway
[56] Renal cell carcinoma
[57] cGMP-PKG signaling pathway
[58] Dopaminergic synapse
[59] Oxytocin signaling pathway
[60] Endometrial cancer
[61] Hypertrophic cardiomyopathy (HCM)
[62] cAMP signaling pathway
[63] Hippo signaling pathway
[64] Choline metabolism in cancer
[65] Dilated cardiomyopathy (DCM)
[66] mTOR signaling pathway
[67] Cholinergic synapse
[68] TGF-beta signaling pathway
[69] GnRH signaling pathway
[70] Adipocytokine signaling pathway
[71] Basal cell carcinoma
[72] Cushing syndrome
[73] Melanogenesis
[74] Hepatocellular carcinoma
[75] Adherens junction

Supplemental Figure S7. **Comparisons of sample fractions in subgroups of fibroproliferative samples in MPH dataset.** The top panel is faction of dcSSc samples (white) in each group. The bottom panel is the fraction of disease stage in each group (white is early, and grey is late).

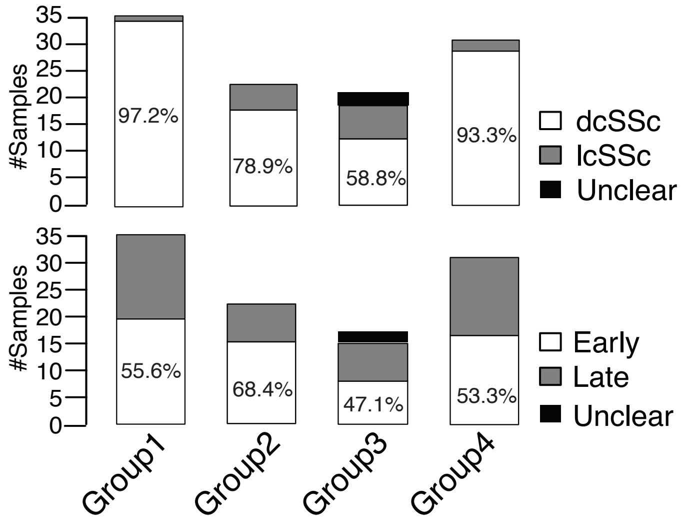

Supplemental Figure S8. **Tracks of samples' pulmonary fibrosis measurements in Hinchcliff dataset.** Different shapes infer four pulmonary fibrosis measurements and different colors suggest different samples with complete data along time points. A) For the only sample classified in group 2 (high FOXO4 activity and low Core binding factor activity). B) For three samples classified in group 4 (low FOXO4 activity and high Core binding factor activity). There is no samples with complete data classified in group 3 (low FOXO4 activity and low Core binding factor activity).

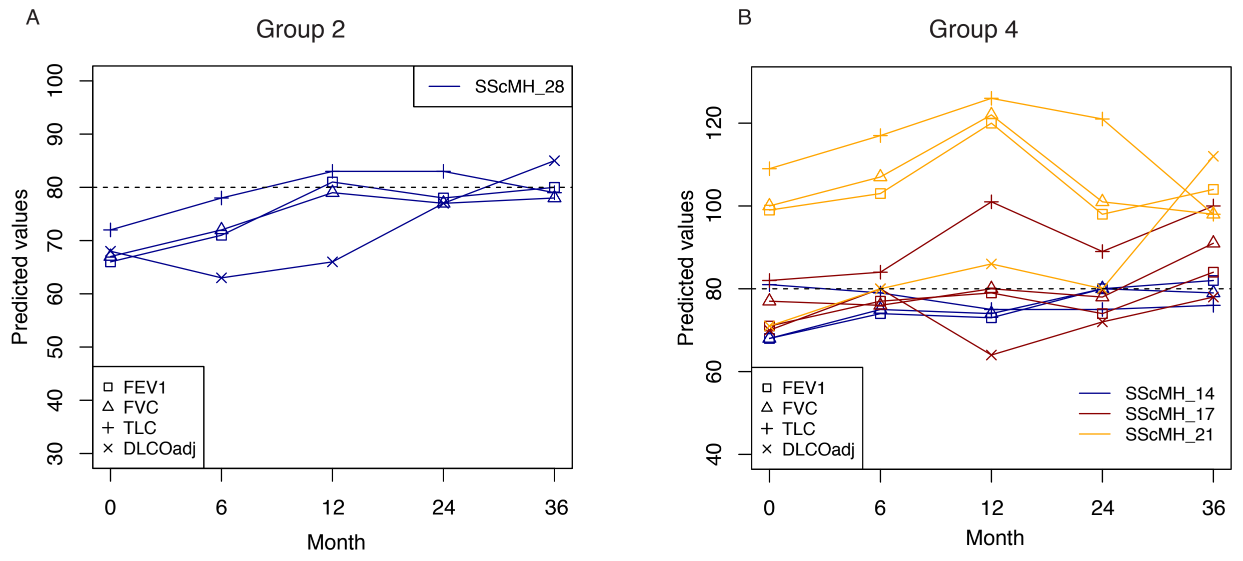

Supplemental Figure S9. **Validation of ILD regulator pair in PBMC dataset.** A) Samples distributions of groups identified with FOXO4 and core binding factor. B) ILD fractions for groups. Fractions are listed below the barplot.

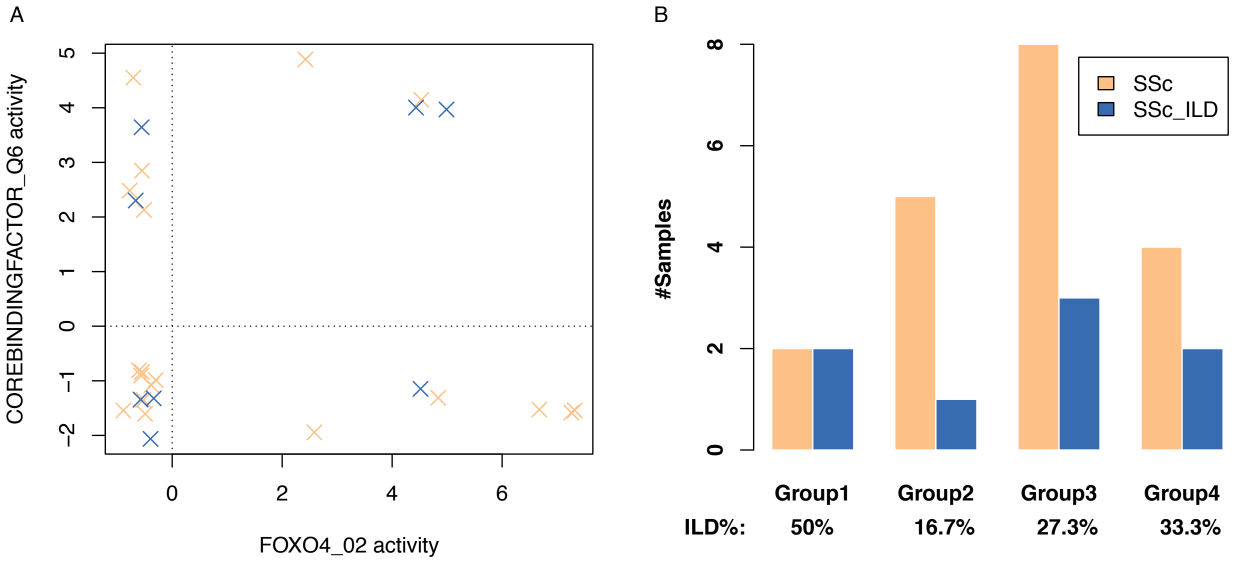

Supplemental Figure S10. **Regulator-target network in inflammatory subset.** Red nodes represent regulators whose activity scores are positively correlated with MRSS and cyan nodes represent regulators whose negatively scores are positively correlated with MRSS. The size of the node is positively correlated to its degree of connection. The arrow direction points from regulator to target and a circle means self-regulation. Here, we want to highlight the RUNX1-related core binding factor.

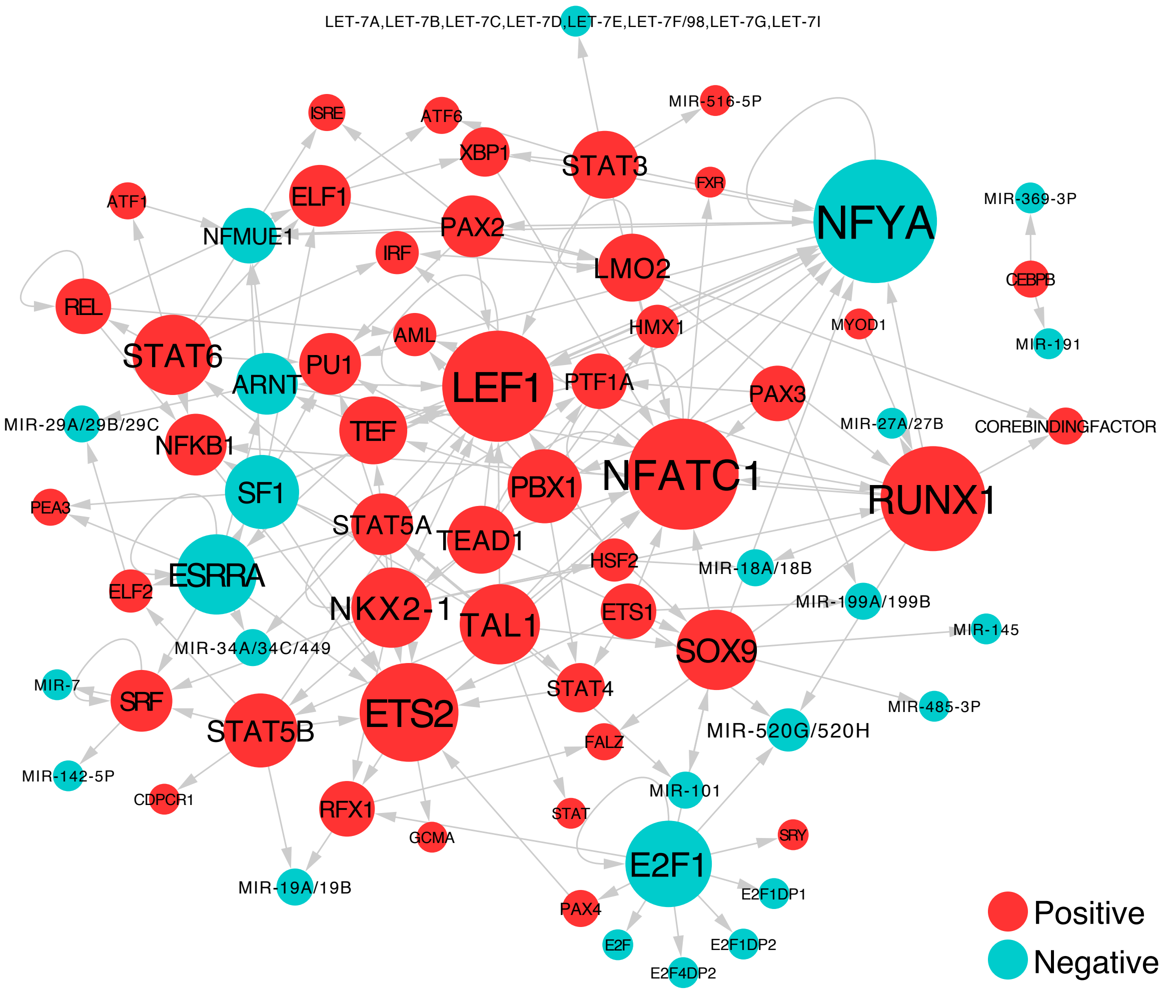

Supplement: Supplemental data [file jciinsight-5-137567-s149.pdf]
